# Early mesodermal development in the patellogastropod *Lottia goshimai*

**DOI:** 10.1111/eva.13373

**Published:** 2022-04-14

**Authors:** Dehui Sun, Pin Huan, Baozhong Liu

**Affiliations:** ^1^ CAS and Shandong Province Key Laboratory of Experimental Marine Biology Institute of Oceanology Chinese Academy of Sciences Qingdao China; ^2^ University of Chinese Academy of Sciences Beijing China; ^3^ Laboratory for Marine Biology and Biotechnology Pilot National Laboratory for Marine Science and Technology (Qingdao) Qingdao China

**Keywords:** ectomesoderm precursor, *Lottia*, mesoderm, *snail* gene, Spiralia

## Abstract

Mesodermal development is essential to explore the interlineage variations in the development of spiralians. Compared with model mollusks such as *Tritia* and *Crepidula*, knowledge about the mesodermal development of other molluscan lineages is limited. Here, we investigated early mesodermal development in the patellogastropod *Lottia goshimai*, which shows equal cleavage and has a trochophore larva. The endomesoderm derived from the 4d blastomere, that is, the mesodermal bandlets, was situated dorsally and showed a characteristic morphology. Investigations of the potential mesodermal patterning genes revealed that *twist1* and *snail1* were expressed in a proportion of these endomesodermal tissues, while all of the five genes we investigated (*twist1*, *twist2*, *snail1*, *snail2*, and *mox*) were expressed in ventrally located ectomesodermal tissues. Relatively dynamic *snail2* expression suggests additional roles in various internalization processes. By tracing *snail2* expression in early gastrulae, the 3a^211^ and 3b^211^ blastomeres were suggested to be the precursors of the ectomesoderm, which elongated to become internalized before division. These results help to understand the variations in the mesodermal development of different spiralians and explore the different mechanisms by which ectomesodermal cells are internalized, which has important evolutionary implications.

## 1 INTRODUCTION

Spiralians comprise one of the three major clades of bilaterians, which include various animal clades with extremely diverse body plans, such as mollusks, annelids, nemerteans, and platyhelminthes (Hejnol, 2010; Henry, 2014; Kocot, 2016). Despite this diversity in adult body plans, spiralian development shows remarkable conservation, such as the stereotypic spiral cleavage and the D‐quadrant organizer (Henry, 2014; Henry & Martindale, 1999; Lambert, 2008; Nielsen, 2010). In particular, the highly regular spiral cleavage makes it feasible to compare development at the single‐cell level, even among phylogenetically distant species.

Such comparisons reveal that the D‐quadrant blastomeres play unique roles in spiralian development. The well‐known spiralian organizer, referring to the blastomere(s) that induces the formation of the second body axis and the development of multiple organs, is derived from the D‐quadrant lineage (Henry, 2014; Lambert, 2008; Seaver, 2014). Another unique D‐quadrant blastomere is 4d, which is formed in the sixth cleavage and shows a tight correlation with the organizer in some spiralians (especially mollusks). Specifically, the 4d blastomere is a common precursor of mesoderm in most spiralians (Henry & Martindale, 1999; Lambert, 2008). Thus, understanding mesodermal development derived from 4d may be essential to explore the origin of spiralians and to understand how the highly conserved genesis of the mesoderm produces diverse adult body plans. The 4d blastomere has been paid particular attention in the study of spiralian development (Henry, 2014; Lambert, 2008). It divides bilaterally to generate the bilateral embryonic tissue, the mesodermal bandlets (Barak et al., 2005; Hejnol et al., 2007; Smith, 1935). The cleavage patterns of 4d have been described in exceptional detail in several spiralians, including the annelids *Platynereis dumerilii*, *Tubifex tubifex*, *Helobdella* sp. Austin and the mollusks *Crepidula fornicata* and *C. convexa* (Fischer & Arendt, 2013; Gline et al., 2011; Lyons et al., 2012; Özpolat et al., 2017).

Despite some exceptions, such as some annelids, 4d produces the mesoderm and the endoderm (Henry & Martindale, 1999; Lambert, 2008; Nielsen, 2010), and thus the 4d‐derived mesoderm could be designated as the endomesoderm. Moreover, the spiralian mesoderm has an additional source. This part of the mesoderm is derived from the second or third micromeres (Henry & Martindale, 1999) and is designated the ectomesoderm, since their precursors also generate ectoderm. Spiralian ectomesoderm exhibits some unusual characteristics and is emphasized in spiralian development (Hejnol, 2010; Martín‐Durán & Marlétaz, 2020). In contrast to the relatively stable origin of the endomesoderm, the precursors of the ectomesoderm vary (Henry & Martindale, 1999; Lyons & Henry, 2014), but an ancestral origin from 3a and 3b blastomeres is suggested (Lyons & Henry, 2014). The internalization of ectomesoderm has been clearly described in *Crepidula* (Lyons et al., 2015). Genes involved in ectomesodermal development have also been explored (Osborne et al., 2018; Wu & Lambert, 2021).

Modifications of developmental procedures have been frequently observed in spiralians, which are clearly lineage‐specific variations and have contributed to the diversity of spiralians (Henry, 2014; Henry & Martindale, 1999; Lambert, 2010; Nielsen, 2010; Seaver, 2014). For mollusks, various lineages have adopted very different developmental modes (Henry, 2014), for example, equal or unequal cleavage (referring to whether the first two zygotic divisions are equal) and direct or indirect development (the latter could further diverge with the development of different larval types, such as trochophore and veliger). For instance, the model mollusks *Tritia obsoleta* (also known as *Ilyanassa obsoleta*) and *Crepidula fornicata* both belong to the caenogastropod and develop veliger larvae, but the former exhibits unequal cleavage, while the latter is essentially an equal cleaver (Goulding & Lambert, 2016; Henry & Lyons, 2016). Other gastropods, such as patellogastropods (e.g., *Patella vulgata*) and some vetigastropods (e.g., *Haliotis tuberculata*), perform equal cleavage but exhibit a trochophore stage before the development of veliger larvae (Smith, 1935; van den Biggelaar, 1993). In accordance with their varied developmental strategies, the early development of these species exhibits remarkable variations (Chan & Lambert, 2014; Dictus & Damen, 1997; Hejnol et al., 2007; Lyons et al., 2015; van den Biggelaar, 1977; van den Biggelaar, 1993). Investigating these different developmental modes would be important to understand the conservation and variations during spiralian development.

Studies in model mollusks such as *Tritia* and *Crepidula* have revealed many aspects of molluscan mesodermal development (Chan & Lambert, 2014; Hejnol et al., 2007; Lyons et al., 2012, 2015; Osborne et al., 2018; Perry et al., 2015; Wu & Lambert, 2021). Nevertheless, investigations of other lineages may provide additional information for interlineage comparisons. In particular, while *Tritia* and *Crepidula* do not develop trochophore larvae, understanding the mesodermal development in mollusks with a trochophore stage would be especially informative. This is because trochophore larvae also develop in other spiralians (such as annelids) and thus are evolutionarily important. In contrast, veliger larvae are confined to mollusks (mainly gastropods and bivalves) and are suggested to be a modified form of trochophore (Nielsen, 2018). Indeed, investigations of the expression of several mesodermal patterning genes in the patellogastropod *Patella vulgata* have revealed considerable lineage‐specific characteristics that are important to understand the body plan of a trochophore larva (Lespinet et al., 2002; Nederbragt et al., 2002).

To reveal more information on the mesodermal development of mollusks with a trochophore stage, in the present study, we investigated the mesodermal development of another patellogastropod species, *Lottia goshimai*, from the onset of gastrulation (when 4d was born) to the early trochophore stage. The morphological characteristics of the suspected 4d lineage, as well as the expression of several potential mesodermal marker genes, were explored. In particular, we determined the presumptive precursors for the ectomesoderm of the species (at least for a proportion of them) to be 3a^211^ and 3b^211^. These results add to the knowledge of molluscan/spiralian mesodermal development.

## 2 MATERIALS AND METHODS

### 2.1 Animals and sample collection

Adults of *L. goshimai* were collected from intertidal rocks in Qingdao, China. Gamete collection and artificial fertilization were conducted as described previously (Tan et al., 2022). Fertilized oocytes were cultured in filtered seawater (FSW) containing 100 unit/mL benzylpenicillin and 200 μg/ml streptomycin sulfate at 25°C in an incubator. Developmental stages are referred to as hours postfertilization (hpf). At the desired developmental stages, the embryos were fixed in 4% paraformaldehyde (PFA) (1 × PBS, 100 mM EDTA, 0.1% Tween‐20, pH 7.4) overnight at 4°C. For whole mount in situ hybridization (WMISH), the embryos were transferred to methanol and stored at −20°C. For phalloidin staining, the embryos were transferred to PBST (1 × PBS, 0.1% Tween‐20, pH 7.4) and stored at 4°C. The embryos for scanning electron microscopy (SEM) were fixed in 2.5% glutaraldehyde at 4°C overnight.

### 2.2 Sequence cloning and phylogenetic analyses

The candidate mesodermal patterning genes (*twist* and *snail*) were retrieved from a developmental transcriptome of *L. goshimai*. Phylogenetic analyses were performed using maximum likelihood (ML) methods with the MEGA 6.0 package (http://www.megasoftware.net/). Complete amino acid sequences were used in the analyses.

### 2.3 Whole mount in situ hybridization, phalloidin staining, and scanning electron microscopy

The primers shown in Table S1 were used to generate probes for WMISH. Specifically, the T7 promoter sequence (taatacgactcactataggg) was included in the 5′ upstream of the reverse primers to produce PCR products suitable for RNA probe synthesis through in vitro transcription. WMISH (Huan et al., 2020), phalloidin staining (Kurita et al., 2009), and SEM (Tan et al., 2017) were performed as described previously.

### 2.4 Microscopy

Images were recorded using a Nikon 80i microscope or an LSM 710 laser‐scanning confocal microscopy system (ZEISS, Germany).

## 3 RESULTS

### 3.1 Overall development of *L. goshimai*

**FIGURE 1:**
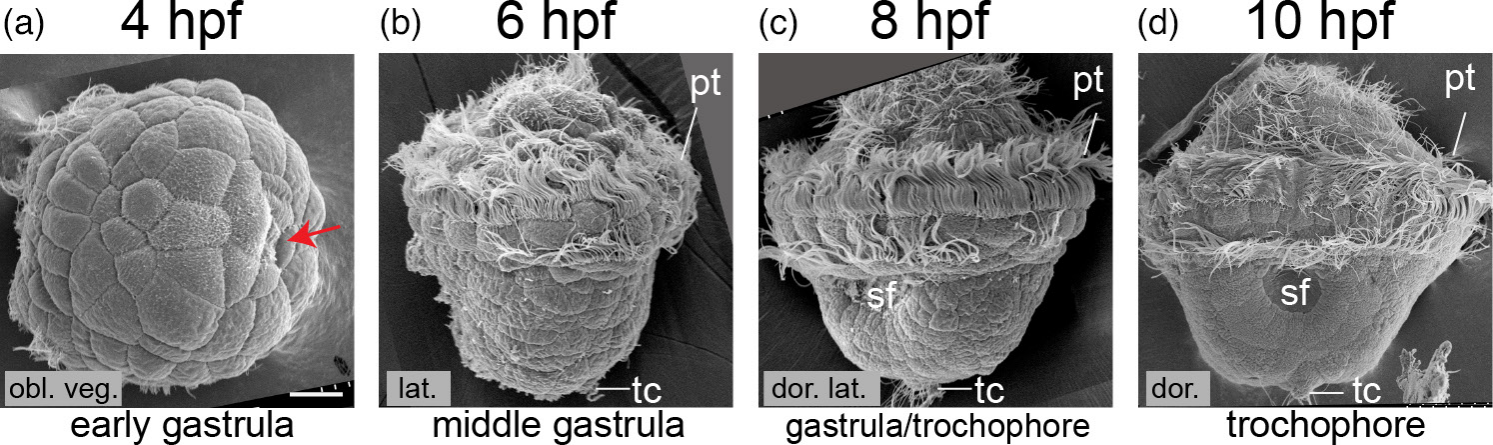
Overall development of *Lottia goshimai*. Panel a is an oblique vegetal view of an early gastrula (4 hpf). The red arrow indicates the small depression caused by the invagination of 4d descendants, which should be the earliest morphogenetic change during gastrulation. Panels b and c show middle and late gastrulae. Panel d shows a trochophore. pt, prototroch; sf, shell field; tc, terminal cell. The bar represents 25 μm

The early development of *L. goshimai* is highly similar to that of *Patella*. Figure 1 shows representative images of samples from the early gastrula to the trochophore. Generation of the 4d blastomere, which represents the earliest specification of the mesoderm, occurred at the 64‐cell stage (approximately 3.5 hpf). It then divided and invaginated, marking the onset of gastrulation (Figure 1a). Gastrulation completed in the subsequent four hours, and a trochophore larva emerged at approximately 8–9 hpf (Figure 1b,c). At 10 hpf, the early trochophore developed, possessing a larval shell plate on the dorsal side, a ventral plate and a larval mouth on the ventral side, and terminal cells at the vegetal pole (Figure 1d). The molluscan mesoderm was specified during gastrulation. We, therefore, focused on the period of gastrulation (3.5–8 hpf), while a few trochophore larval stages (9–10 hpf) were included.

### 3.2 Development of (endo)mesoderm during gastrulation

**FIGURE 2:**
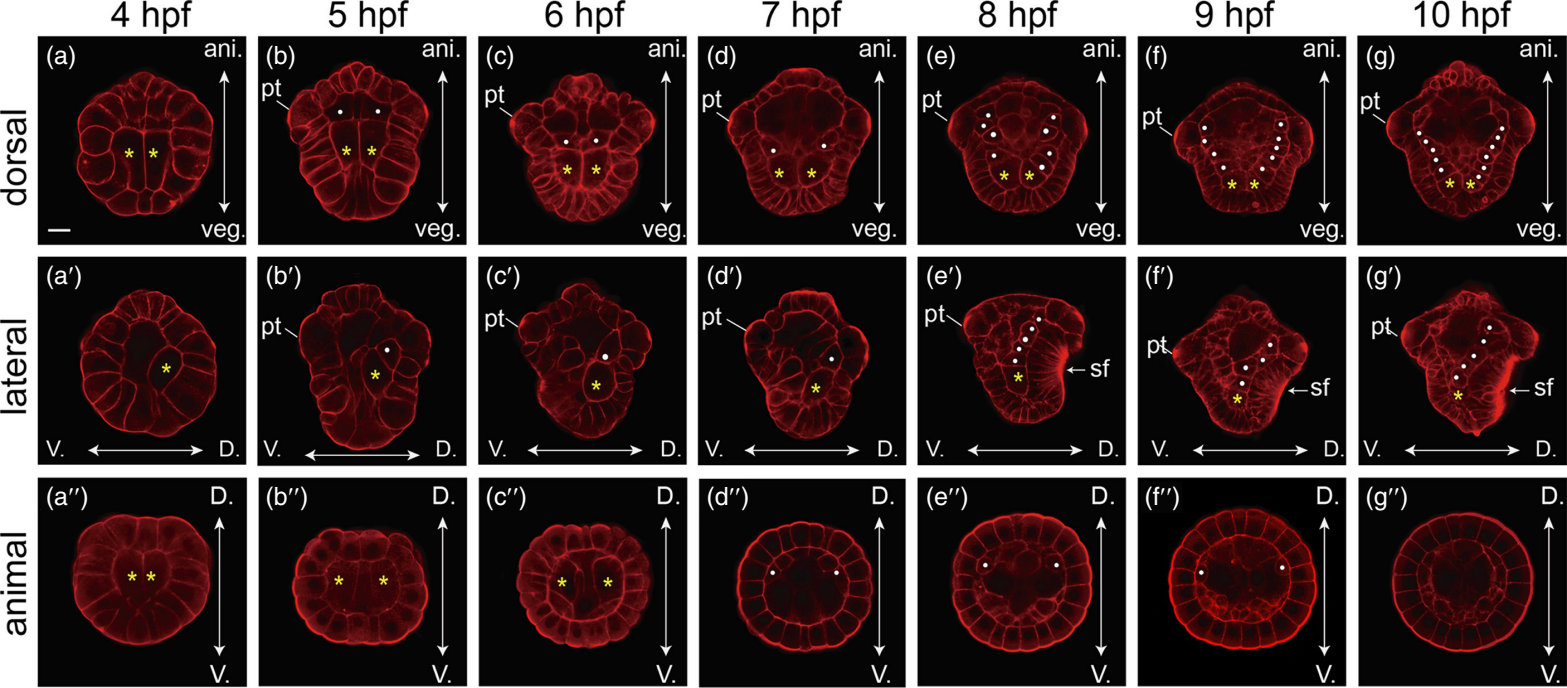
Development of mesodermal bandlets in *Lottia goshimai*. All panels are CLSM (confocal laser‐scanning microscopy) optical sections. Panels a‐g are dorsal views with animal on the top; a′‐g′ are lateral views with dorsal to the right and animal on the top; a″‐g″ are animal views with dorsal on the top. The embryos were stained with fluorescence‐labeled phalloidin to show the outlines of individual cells. The suspected mesodermal bandlets are labeled using yellow asterisks (the terminal cells) and white dots (other cells). Note that although we labeled two bilateral cells in d″‐f″, they may not correspond to the cells shown in the upper rows since it was difficult to discriminate between the various cells with similar morphological characteristics in the sections. ani., animal; veg., vegetal; V., ventral; D., dorsal; pt, prototroch; sf, shell field. The bar represents 25 μm

As previously described, the spiralian 4d blastomere divides bilaterally to produce mesodermal bandlets that exhibit characteristic morphological features (Fischer & Arendt, 2013; Lyons et al., 2012; Smith, 1935). We thus first traced the development of 4d‐derived cells using phalloidin staining. The division of 4d generated paired mesentoblasts (left of the midline (ML) and right of the midline (MR)), which were easily recognizable from a 4 hpf embryo (Figure 2a,a″). These two cells were close to the dorsal ectodermal cells (Figure 2a’) and likely gave rise to the symmetrically distributed mesodermal bandlets in subsequent development (Figure 2). Notably, although we observed two bilaterally organized bandlets inside the embryos, and they showed strong associations with 4d, we could not precisely determine if all of the cells of the bandlets were derived from 4d or if they contained all of the 4d descendants since we only recognized the bandlets based on their morphological characteristics. In the subsequent text, we will generally consider that they are derived from 4d, despite some uncertainties (see more information in the Discussion). Moreover, although we used the term “mesentoblast” here and “endomesoderm” in subsequent descriptions to refer to 4d and its descendants, it is not known whether 4d generates endoderm in *L. goshimai*. Nevertheless, given that 4d producing both endoderm and mesoderm is observed in model gastropods such as *Tritia* and *Crepidula* (Chan & Lambert, 2014; Hejnol et al., 2007), we think utilizing these commonly used terms would help with interspecies comparisons, while we urge a cautious attitude.

At 5 hpf, the locations of ML and MR changed slightly and were more adjacent to the animal pole, likely related to the invagination of the endodermal tissues (macromeres) and the axial elongation of the embryo (Figure 2b). The two mesentoblast cells experienced one round of division at this stage. The divisions were along the animal–vegetal (AV) axis, producing two pairs of mesodermal cells; the top of the mesodermal tissues reached the prototroch (Figure 2b’). From 5 hpf to 7 hpf, we did not observe an obvious increase in the cell numbers of these mesodermal tissues (Figure 2b′‐d′). In contrast, the tissues exhibited rapid proliferation shortly afterward (Figure 2d′‐e′). In just one hour (from 7 hpf to 8 hpf), a pair of characteristic mesodermal bandlets were formed, comprising 4–5 pairs of cells, and they were aligned beneath the dorsal ectoderm (Figure 2e,e′). No other major changes occurred for these mesodermal bandlets until the last time point that was investigated (10 hpf), except that the middle part of the tissues gradually bent toward the ventral side (Figure 2f′,g′) due to the invagination of the shell field. Notably, during the whole period investigated (4–10 hpf), the most posterior cell was always larger than the other cells inside each mesodermal bandlet (the yellow asterisks in Figure 2b′–g′).

### 3.3 Expression of mesodermal marker genes in early development

We next explored the expression dynamics of several mesodermal patterning genes, including *twist*, *snail*, and *mox*. These genes participate in the mesodermal development of various animals such as *Caenorhabditis elegans*, *Drosophila melanogaster*, *Xenopus laevis*, and *Mus musculus* (Candia et al., 1992; Castanon & Baylies, 2002; Nieto, 2002; Sandmann et al., 2007). A *mox* homolog was previously identified in *L. goshimai* (Wang et al., 2019). Blast searching against the developmental transcriptome revealed two *twist*‐like and two *snail*‐like genes, and phylogenetic analyses confirmed their orthologies (Figure S1). The existence of two *snail* genes has been observed in both vertebrates (in humans and mice) and patellogastropods (in *Patella* and *Lottia*), but our results do not indicate that they are derived from a common duplication event (Figure S1b). In contrast, the existence of an additional *twist* gene seems to be a lineage‐specific characteristic of *L. goshimai* (Figure S1a).

We did not detect *twist2* expression in most of the stages we investigated (except for restricted and very weak expression in 10 hpf trochophores; Figure S2); thus, we did not include this gene in most cases in the subsequent analysis. The other four genes (*twist1*, *snail1*, *snail2*, and *mox*) all showed distinct expression patterns during the period investigated. Notably, although these genes are often involved in mesodermal development, roles in nonmesodermal tissues have been reported (Nieto, 2002). Therefore, we suggest a cautious attitude when assigning an expression region to be mesodermal in nature (see more information in the Discussion).

#### 3.3.1 Marker gene expression in the suspected endomesoderm

**FIGURE 3:**
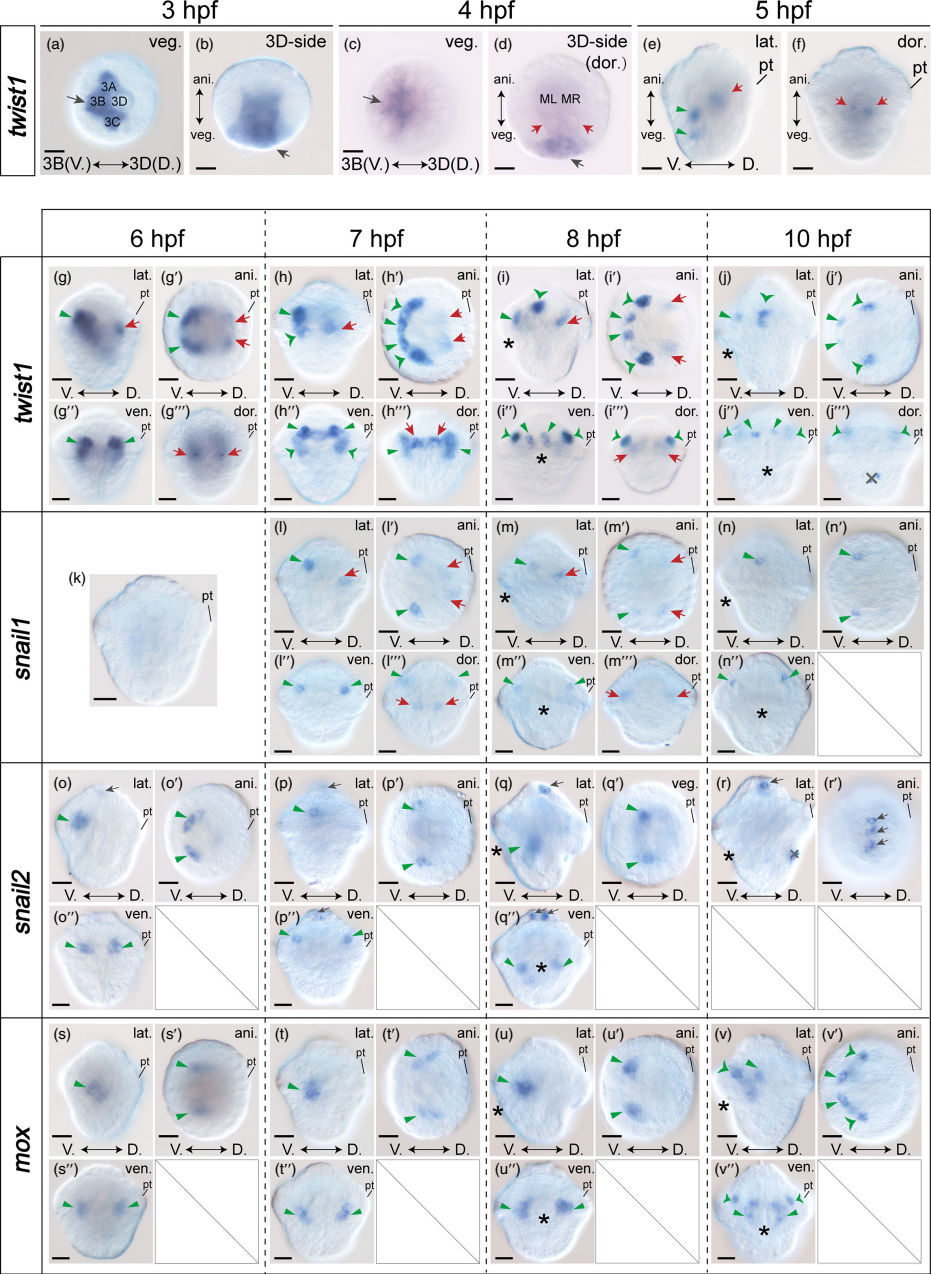
Expression of mesodermal marker genes of *Lottia goshimai*. Panels a‐f show *twist1* expression in early embryos (3–5 hpf). Panels g‐v show marker gene expression from 6 to 10 hpf. For each stage, lateral (lat.), ventral (ven.), dorsal (dor.), and animal (ani.) views are shown when they show WMISH signals. The green arrowheads and red arrows indicate the expression in suspected ectomesodermal and endomesodermal cells, respectively. When the expression was scattered, different types of arrowheads are used to indicate different parts of the expression and to show the corresponding expression in different panels (e.g., in h, h′, h″ and h‴). The gray arrows indicate nonmesodermal expression, and the gray crosses indicate nonspecific staining (r). The asterisks indicate the blastopore. V., ventral; D., dorsal; pt, prototroch. Bars represent 25 μm

The mesodermal bandlets are derived from the 4d blastomere and are referred to as endomesoderm. We first focused on the expression of marker genes in these tissues. Somewhat unexpectedly, we only detected rather limited marker gene expression in the suspected endomesodermal tissues. At 4 hpf, two bilateral expression regions of *twist1* could be detected, coinciding with the ML and MR blastomeres (red arrows in Figure 3d). This is the sole marker gene expression that could be readily determined in the endomesodermal tissues. In the subsequent development (5–8 hpf), bilateral *twist1* expression that generally coincided with the mesodermal bandlets was sustained (red arrows in Figure 3e‐i). However, *twist1* expression was never detected in the entire bandlets but was only observed in a few bilaterally organized cells adjacent to the prototroch (red arrows in Figure 3e,f,g′‐i′); it became undetectable at 10 hpf (Figure 3j′). Another gene that may be expressed in endomesodermal tissues is *snail1*. Some *snail1* expression, also bilaterally distributed, was detected in dorsal internal tissues that coincided with the mesodermal bandlets at 7 and 8 hpf (the red arrows in Figure 3l,m).

#### 3.3.2 Marker gene expression in the suspected ectomesoderm

Most of the other expression patterns was detected in internal tissues on the ventral side. These expression regions were spatially close in general, indicating that they may together mark another proportion of mesodermal tissues, that is, the ectomesoderm. This notion is consistent with a previous report suggesting *mox* to be an ectomesodermal marker in the gastropod *Haliotis* (Hinman & Degnan, 2002). The *mox* expression was explored in 10‐ and 12‐hpf trochophores of *L. goshimai* previously, and its correlations with the foot anlagen were also revealed (Wang et al., 2019). Moreover, we found that the expression of *twist1* and *snail2* could be detected in early gastrulae (5 hpf and before), and it likely indicated the internalization of ectomesodermal cells. This part of the results will be presented later, and here, we only describe the expression from 6 to 10 hpf.

The expression of the known ectomesodermal marker gene *mox* was detectable beginning at 6 hpf (Figure 3s). It was stably detected in two bilateral groups of cells, despite an evident increase in the numbers of stained cells at 10 hpf (Figure 3s–v). Comparably, stable patterns were observed for the ventral expression of *twist1* (green arrowheads in Figure 3g–j). At 6 hpf, it was detected in two bilateral groups of cells adjacent to the prototroch (green arrowheads in Figure 3g). It transited into a semicircular pattern at 7 hpf and became somewhat scattered at 8 and 10 hpf (green arrowheads in Figure 3h–j). The ventral expression of *snail1* was detected in two subepidermal cells from 7 to 10 hpf (green arrowheads in Figure 3l–n). The only detectable *twist2* expression during the stages we investigated (in the 10 hpf trochophore) was highly similar to *snail1* expression (Figure S2b; compare it to Figure 3n′).

In contrast to the relatively stable expression of other genes, *snail2* expression was dynamic. At 6 hpf, *snail2* expression probably coincided with the expression regions of *twist1* (Figure 3o, compare to Figure 3g). However, this expression shut down later, and *snail2* expression was detected in two cells beneath the prototroch at 7 hpf, highly similar to the ventral part of *snail1* expression at this stage (Figure 3p, compare it to Figure 3l). Then, the expression of *snail2* in this region decreased again; at 8 hpf, *snail2* expression was detected in two groups of ventral cells posterior to the blastopore (Figure 3q). At 10 hpf, no *snail2* expression in internal tissues was detectable (Figure 3r).

#### 3.3.3 Expression in nonmesodermal tissues

**FIGURE 4:**
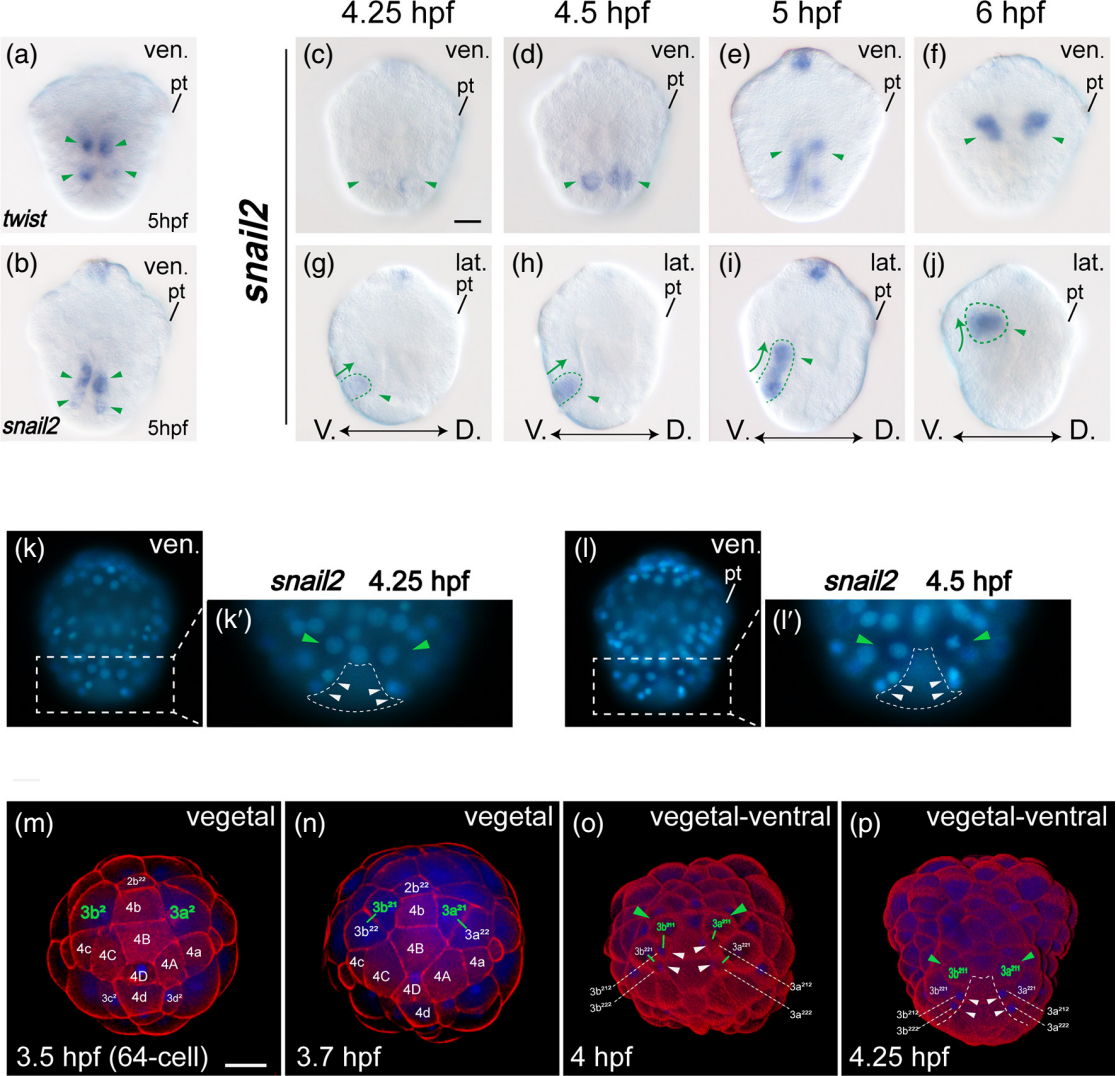
Genesis of the presumptive ectomesoderm. Panels a‐b are ventral views with the animal on the top. Green arrowheads indicate expression in the ectomesoderm. Panels c‐j show the expression of *snail2* from 4.25 hpf to 6 hpf. The green arrowhead indicates the expression of *snail2* in the presumptive ectomesoderm. Panels k‐l show morphological characteristics at the vegetal pole that could be used to trace the *snail2*‐expressing cells (green arrowheads), including the uninternalized endodermal tissues (dashed lines) and two pairs of small cells with characteristic concentrated nuclei (white arrowheads). Panels c and k and d and l are the same embryos, respectively. Panels m‐p show the divisions at the vegetal pole from 3.5 hpf (the 64‐cell stage) to 4.25 hpf, when the earliest *snail2* expression could be detected. The cell lineage leading to the ectomesoderm is labeled green. The short green lines indicate sister‐cell relationships. Compare o, p and k′, l′ to see the common morphological characteristics at the vegetal pole. Bars represent 25 μm

Expression of the marker genes could be detected in nonmesodermal tissues. At the 60‐cell stage (approximately 3 hpf), which was immediately before the beginning of gastrulation (3.5–4 hpf), strong expression of *twist1* was detected in three macromeres, namely 3a, 3b, and 3c (gray arrows in Figure 3a,b). *twist1* expression in the presumptive descendants of 3a–3c was sustained in the 4 hpf embryo (gray arrows in Figure 3c,d). There was constant *snail2* expression in a few apical cells from 4 to 10 hpf (gray arrows in Figures 3o‐r and 4), which were obviously ectodermal.

### 3.4 Internalization of presumptive ectomesodermal cells in early gastrulae

The spatially close expression of *twist1* (the ventral proportion), *twist2* (only detectable at 10 hpf), *snail1* (the ventral proportion), *snail2*, and *mox* suggested that these genes were all expressed in ectomesodermal tissues. We found that although *mox* expression was only detectable since 6 hpf, *twist1* and *snail2* were expressed in 5 hpf embryos (Figure 4a,b). At this stage, highly consistent expression of the two genes, probably overlapped, was detected in cells that were mostly internalized but still partially exposed (see the details later). Given that the expression region of each gene at 6 hpf was spatially close to that at 5 hpf (e.g., compare Figure 3e and g), it is reasonable to assume that the cells expressing the two genes gradually migrated into the interior of the embryo. In other words, this assumed process may reflect the internalization of early ectomesodermal cells.

We thus explored earlier samples to reveal more details of this process. Given that the expression of *twist1* and *snail2* was probably overlapped in ventral cells at 5 hpf (Figure 4a,b), we used *snail2* in subsequent analysis. We collected samples between 4 and 5 hpf, aiming to trace the dynamics of *snail2* expression during this period. The results revealed the earliest *snail2* expression in two bilateral ventral cells at 4.25 hpf (Figure 4c,g). At 4.5 hpf, in addition to evident gene upregulation (Figure 4d), the cells became elongated (Figure 4h). The elongation became much more evident at 5 hpf (Figure 4i), and the two cells experienced one round of division during this period (Figure 4e). Interestingly, the divisions of the two cells were not synchronous: the left cell always divided slightly earlier than the right cell (Figure 4e). Together, these results indicate that these two cells initiated *snail2* expression at 4.25 hpf and then became elongated and were gradually internalized in the subsequent 2 h.

We next sought to determine the identity of the earliest *snail2*‐expressing cells, which emerged at 4.25 hpf (Figure 4c,k). It was not possible to directly identify the two cells at this stage due to the lack of a cell lineage map. Fortunately, we found that the two cells were situated on both sides of the uninternalized endodermal tissues, anterior to two pairs of small cells with concentrated nuclei (Figure 4k′,l′). These morphological characteristics served as ideal landmarks. We then traced the divisions of the cells at the vegetal pole from the 64‐cell stage to the time point when the two target cells emerged. These results clearly revealed that the two cells were derived from the 3a^2^ and 3b^2^ blastomeres, namely 3a^211^ and 3b^211^ (Figure 4m‐p).

## 4 DISCUSSION

Mesodermal development, with special focus on the 4d blastomere and ectomesoderm, has always been a major topic of study on spiralian development. Various spiralian representatives, including mollusks, annelids, brachiopods, and platyhelminthes, have been investigated, and the results have revealed both conservation and remarked interlineage variations (Andrikou et al., 2019; Chan & Lambert, 2014; Dill et al., 2007; Girstmair & Telford, 2019; Gline et al., 2011; Hejnol et al., 2007; Hinman & Degnan, 2002; Lespinet et al., 2002; Martín‐Durán et al., 2017; Passamaneck et al., 2015; Pfeifer et al., 2014). In the present study, we investigated early mesodermal development in the patellogastropod mollusk *L. goshimai* based on confocal laser‐scanning microscopy and the expression of potential mesodermal patterning genes. The results describe the development of endomesodermal and ectomesodermal tissues on the dorsal and ventral sides, respectively. Overall, the mesodermal development of *L. goshimai* shows similarities to other spiralians and exhibits lineage‐specific characteristics that may be related to the developmental mode of the species (equal cleavage and having a trochophore larva) (see below). These results will help us understand the interlineage variations in the mesodermal development of mollusks and spiralians and explore the evolutionary implications. On the contrary, given that determining a mesodermal fate requires cell lineage analysis, which is not available in *L. goshimai* at present, we declare a cautious attitude and firmly state that our results and related speculations should be verified by cell lineage analysis in future.

### 4.1 4d blastomere and the mesodermal bandlets

The fact that 4d blastomeres generate endomesoderm is conserved in almost all spiralian lineages. 4d divides bilaterally and produces bilateral mesodermal bandlets (Fischer & Arendt, 2013; Gline et al., 2011; Lyons et al., 2012; Özpolat et al., 2017). This is what we observed in *L. goshimai* (Figure 2), and we found that the mesodermal bandlets were morphologically similar to those of most other spiralians. Specifically, they were bilaterally situated on the dorsal part of the embryo, beneath the ectoderm. From the dorsal view, the two bandlets exhibit a V‐shape (Figure 2d–g). Investigations of the 4d cleavage patterns in two spiralians, the mollusk *Crepidula* and the annelid *Platynereis*, all reveal that ML and MR divisions follow stem cell‐like behaviors, with new cells budded off along the animal–vegetal axis (Fischer & Arendt, 2013; Lyons et al., 2012; Özpolat et al., 2017). We found that the terminal cell of each mesodermal bandlet was always the largest cell, indicating a similar stem cell‐like behavior during the genesis of mesodermal bandlets in *L. goshimai*.

Without the aid of lineage tracers, we did not obtain sufficient information to determine the cleavage pattern of 4d; this raises several caveats regarding the “mesodermal bandlets” that we recognized (Figure 2a–g). These tissues may only be one part of the 4d‐derived endomesoderm. Indeed, the cells adjacent to bandlets showed morphological characteristics similar to those of the bandlet cells (Figure 2a″–g″). They could also be mesodermal (and/or endodermal) tissues derived from 4d. These potential 4d‐derived, unrecognized endomesodermal tissues may explain the apparent “arrest” of the development of mesodermal bandlets from 5 to 7 hpf. During this period, each recognized bandlet comprised two cells (Figure 2b′–d′), seemingly indicating cell cycle arrest. However, it is also possible that cell divisions during this period occurred in other directions, which caused the daughter cells to diverge from the bandlets that we recognized. Individuals of 4d descendants somewhat departing from the stem of mesodermal bandlets have been clearly revealed in both *Crepidula* and *Platynereis* (Fischer & Arendt, 2013; Lyons et al., 2012; Özpolat et al., 2017).

Another caveat is that the mesodermal bandlets we recognized were not solely derived from 4d, especially in late samples. The cells forming the bandlets increased quickly from 7 to 8 hpf and continued to increase in the subsequent two hours (Figure 2d‐g). Such an increase in cell numbers could be caused by cell divisions. Alternatively, given that the ectomesodermal tissues are developing in this period, it is also possible that some ectomesodermal cells migrate to participate in the bandlets. Active migrations of mesodermal cells over relatively long distances have been revealed in two other gastropod mollusks, *Tritia* and *Crepidula* (Lyons et al., 2015; Wu & Lambert, 2021), and in the bivalve mollusk *Septifer* (Kurita et al., 2016). In particular, some ectomesodermal cells can be spatially close to the 4d clones in *Crepidula* (figure 8 of that study; Lyons et al., 2015).

### 4.2 Gene expression in presumptive mesodermal tissues

We investigated early mesodermal development by exploring the expression of several marker genes (*twist*, *snail*, and *mox*). These genes have received particular attention in studies of various spiralian clades (Andrikou et al., 2019; Dill et al., 2007; Hinman & Degnan, 2002; Kozin et al., 2016; Lespinet et al., 2002; Martín‐Durán et al., 2017; Nederbragt et al., 2002; Osborne et al., 2018; Özpolat et al., 2017; Passamaneck et al., 2015; Perry et al., 2015; Pfeifer et al., 2014; Seudre et al., 2021). Nevertheless, they are also implicated in the development of nonmesodermal tissues (Dill et al., 2007; Kozin et al., 2016; Lespinet et al., 2002; Passamaneck et al., 2015; Perry et al., 2015). Thus, we could not precisely determine the nature of the tissues showing marker gene expression in *L. goshimai*. Given the characteristics of mesodermal tissues and the previous studies on spiralian mesoderm, we considered that the internal tissues showing marker gene expression could generally reflect a mesodermal nature (when there were no other stronger indications). The reliability of these assignments was strengthened when the gene expression regions showed associations with two known landmarks, that is, the mesodermal bandlets in the dorsal tissues (endomesoderm) and the *mox* expression in the ventral tissues (ectomesoderm). Notably, although ectomesodermal gene expression has been reported in various mollusks (Nederbragt et al., 2002; Osborne et al., 2018; Perry et al., 2015), we found that *mox* expression in the presumptive ectomesoderm, with a similar expression pattern previously revealed in *Haliotis* (Hinman & Degnan, 2002), was particularly useful as a reference given that (1) the cleavage patterns and larval morphology are similar between *Haliotis* (van den Biggelaar, 1993) and *Lottia* (our observations), and (2) it was revealed that the relatively constant *Haliotis mox* expression was finally confined to muscular tissues inside the foot (thus confirming a mesodermal fate) (Hinman & Degnan, 2002).

Based on these criteria, our results seem to indicate that the mesodermal tissues of *Lottia* generally comprise two parts. One part of the tissue is located in dorsal tissues and generally coincides with the mesodermal bandlets; we thus roughly determined these tissues to be endomesodermal tissues. We detected *twist1* and *snail1* expression in these tissues. Nevertheless, we found that the expression of any one of the two genes was constantly detected in two bilateral cells at a given stage, indicating that they only marked subsets of the mesodermal bandlets. The lack of a gene expressed in the entire mesodermal bandlets makes it impossible to propose a universal endomesodermal marker. In previous studies, similar *snail1* expression patterns in dorsal internal tissues were observed in *Patella*, although the researchers did not propose endomesodermal expression (Lespinet et al., 2002). Endomesodermal expression of *twist* is also observed in the annelids *Alitta* and *Platynereis* (Kozin et al., 2016; Pfeifer et al., 2014) and the brachiopods *Terebratalia* and (possibly) *Novocrania* (Martín‐Durán et al., 2017; Passamaneck et al., 2015). In contrast, *twist* expression is proposed to be confined to ectomesoderm in the gastropod *Patella* (Nederbragt et al., 2002) and the bivalve *Septifer* (Kurita et al., 2016).

The other internal tissues showing marker gene expression are generally distributed ventrally. Given their general spatial adjacency to *mox*‐expressing regions and obvious deviation from the presumptive endomesodermal bandlets, they may be ectomesodermal tissues. Thus, our results indicate that the five genes investigated were all expressed in ectomesodermal tissues. Similar expression profiles in molluscan ectomesoderm have been reported in *Crepidula* (*snail2* and *twist*) (Perry et al., 2015). In *Patella*, as mentioned above, exclusively ectomesodermal *twist* expression has been suggested (Nederbragt et al., 2002). Ectomesodermal *twist* expression has also been revealed in the annelid *Alitta* (Kozin et al., 2016). Thus, the expression of *twist* in both ectomesodermal and endomesodermal tissues has been revealed in the mollusk *Lottia* (this study) and the annelid *Alitta* (Kozin et al., 2016). For the *snail* genes, despite minor variations (e.g., *snail1* expression in the foot epidermis), their expression is highly similar between *Patella* and *Lottia*. Although the researchers did not argue mesodermal expression of *Patella snail* genes (Lespinet et al., 2002), we propose that the ventral *snail1* expression detected in both *Patella* and *Lottia*, which is located in internal tissues, should be in ectomesodermal tissues (Figure 3l–n). *snail2* expression of the two species may also be (ecto)mesodermal (see later).

We found that the presumptive ectomesodermal tissues with marker gene expression were likely nonoverlapping but spatially close (Figure 3), vaguely indicating that there seems to be axial patterning of the related mesodermal tissues. This speculation is reminiscent of the proposal that there may also be ectomesodermal bandlets in mollusks in addition to endomesodermal bandlets (Hinman & Degnan, 2002). In addition, different gene expression profiles in the presumptive ectomesoderm, in parallel with the fact that we only detected restricted expression of *twist1* and *snail1* in a proportion of the presumptive endomesoderm (Figure 3g‐n), indicate that in these early embryos, the mesodermal tissues have differentiated into multiple subpopulations that show distinct molecular patterns. The patterning of early mesoderm is also observed in the mollusk *Crepidula* (Osborne et al., 2018; Perry et al., 2015), the annelids *Capitella* and *Alitta* (Dill et al., 2007; Kozin et al., 2016) and the brachiopods *Terebratalia* and *Novocrania* (Martín‐Durán et al., 2017; Passamaneck et al., 2015).

Among the genes we investigated, a notable fact is that the gene expression regions of *twist1*, *snail1*, and *mox* did not show dramatic differences between adjacent sampling time points, indicating no active behaviors of related cells, which could refer to migration or shape changes. However, this was not the case for *snail2*. The expression of this gene was quite dynamic, showing very different patterns at 6, 7, and 8 hpf (Figure 3o–q). This dynamic could be due to alternative activation of gene expression in different cells and/or active migration of related cells. Given that *snail2* expression seldom overlapped with the expression of other genes, we could not determine the reasons underlying this dynamic expression. It is possible that some *snail2* expression, even in internal tissues, may actually not be in the mesoderm but simply marks the internalization of other types of tissues. This has been proposed for *Patella snail2*, suggesting it has a role in the invagination of the mantle fold (Lespinet et al., 2002).

Conclusively, based on the criteria mentioned above, we determined that most of the internal gene expression regions were mesodermal. These tissues were designated endomesodermal or ectomesodermal, distributed dorsally or ventrally, respectively. This assignment is generally consistent with previous studies, indicating that it is likely correct for most cases. However, as mentioned above, the mesodermal tissues could actively migrate, which may cause mixtures of the two sources of mesoderm. It is also possible that the recognized tissues are not definitely mesodermal and that other types of tissues are internalized.

Last, we noticed the expression of marker genes in obvious nonmesodermal tissues. These include *twist1* expression in 3A–3C blastomeres at the 60‐cell stage (Figure 3a) and *snail2* expression in several apical cells of early embryos (Figure 3o‐r). Apparently, *twist1* expression in macromeres seems to be related to the invagination of these blastomeres, which will occur shortly afterward. Apical *snail2* expression is also observed in *Crepidula* and *Patella*, and its role in the development of neural tissues has been proposed (Lespinet et al., 2002; Perry et al., 2015).

### 4.3 The potential ectomesoderm precursors

The ectomesoderm is emphasized in studies of spiralian development (Kozin et al., 2016; Osborne et al., 2018; Wu & Lambert, 2021). It typically originates from the second and third micromeres, and the exact precursors could vary among different spiralian lineages (Henry & Martindale, 1999; Lambert, 2010). The origin of the ectomesoderm is mainly determined based on cell fate analysis using lineage tracers (Chan & Lambert, 2014; Dictus & Damen, 1997; Hejnol et al., 2007), while the details remain largely unknown regarding how the precursors, which are ectodermal initially, produce mesodermal tissues. Recently, Lyons et al. provided direct evidence of this process by tracing the 3a^2^/b^2^ lineage in fluorescence‐labeled *Crepidula* embryos (Lyons et al., 2015). This study clearly reveals that the descendants of 3a^2^/b^2^ move through the blastopore rim to enter the interior of the embryo, some of which then exhibit mesenchymal characteristics and can migrate over relatively long distances. For patellogastropods, cell fate analysis in *Patella* reveals that 3a, 3b, and 2b blastomeres contribute to the ectomesoderm (Dictus & Damen, 1997), but it is unknown which descendants of the blastomeres produce the ectomesoderm and how they become internalized. Our results revealed that 3a^211^ and 3b^211^ changed their shapes and became internalized during early gastrulation (Figure 4). The relatively stable *twist1* expression indicates that their descendants continue to migrate to get close to the *mox*‐expressing region (it is possible that the *mox*‐expressing cells are also derived from 3a^211^ and 3b^211^). This supports ectomesodermal fates of the cells and generally eliminates the possibility that they are simply internalized ectoderm that form the esophagus wall, given that their distribution patterns do not fit the morphological characteristics of the esophagus and that the locations of the cells are relatively distant from the larval mouth. Collectively, it seems that 3a^211^ and 3b^211^ blastomeres produce ectomesodermal tissues (despite several uncertainties, see below).

Assuming 3a^211^ and 3b^211^ to generate ectomesodermal tissues in *L. goshimai*, it would be interesting to compare their behaviors to those of their counterparts in *Crepidula*, that is, 3a^2^ and 3b^2^ (Lyons et al., 2015). Two major differences are noticed. First, it is clear that when moving into the internal space of the embryo, each 3a^2^/3b^2^ clone of *Crepidula* comprises multiple cells (one can discriminate approximately nine cells in each group from figure 9 of that study; Lyons et al., 2015). In *L. goshimai*, however, it seems that internalization begins before the 3a^211^ and 3b^211^ divisions, that is, this part of the ectomesoderm in *Lottia* starts internalization as single cells (although each of the cells experiences one round of division during internalization). Another difference is that no evident elongation is observed during the internalization of 3a^2^/3b^2^ clones in *Crepidula*. In contrast, evident elongation was observed for internalizing 3a^211^ and 3b^211^ in *Lottia*. These interlineage variations may be related to lineage‐specific developmental modes in the two species (trochophore larvae develop in *Lottia* but not in *Crepidula*; note the evidently different morphological characteristics between the gastrulae of the two species) or other lineage‐specific characters.

Notably, we declare some cautions regarding the term “ectomesoderm precursors.” Since our data are mainly based on WMISH, we could not determine whether the cells with *snail2* (and likely *twist1*) expression had mesodermal fates. In addition, ectomesodermal tissues derived from 2b have been suggested in the patellogastropod *Patella* (Dictus & Damen, 1997), but we have not yet noticed the expression of the marker genes or a comparable internalization process in the suspected 2b clone.

## CONFLICT OF INTEREST

The authors declare no conflict of interest.

## Supporting information

Supplementary Material

## Data Availability

The sequences of the *Lottia goshimai* genes used in this work are deposited in GenBank with accession numbers OL457652‐OL457656.
