# Supplementary material for: Early mesodermal development in the patellogastropod Lottia goshimai
Source: Evol Appl. 2022 Apr 14;16(2):250–61. doi: 10.1111/eva.13373 (PMC9923484; doi:10.1111/eva.13373)
Supplement: Supplementary file 1 — Supplementary Material [file EVA-16-250-s001.pdf]

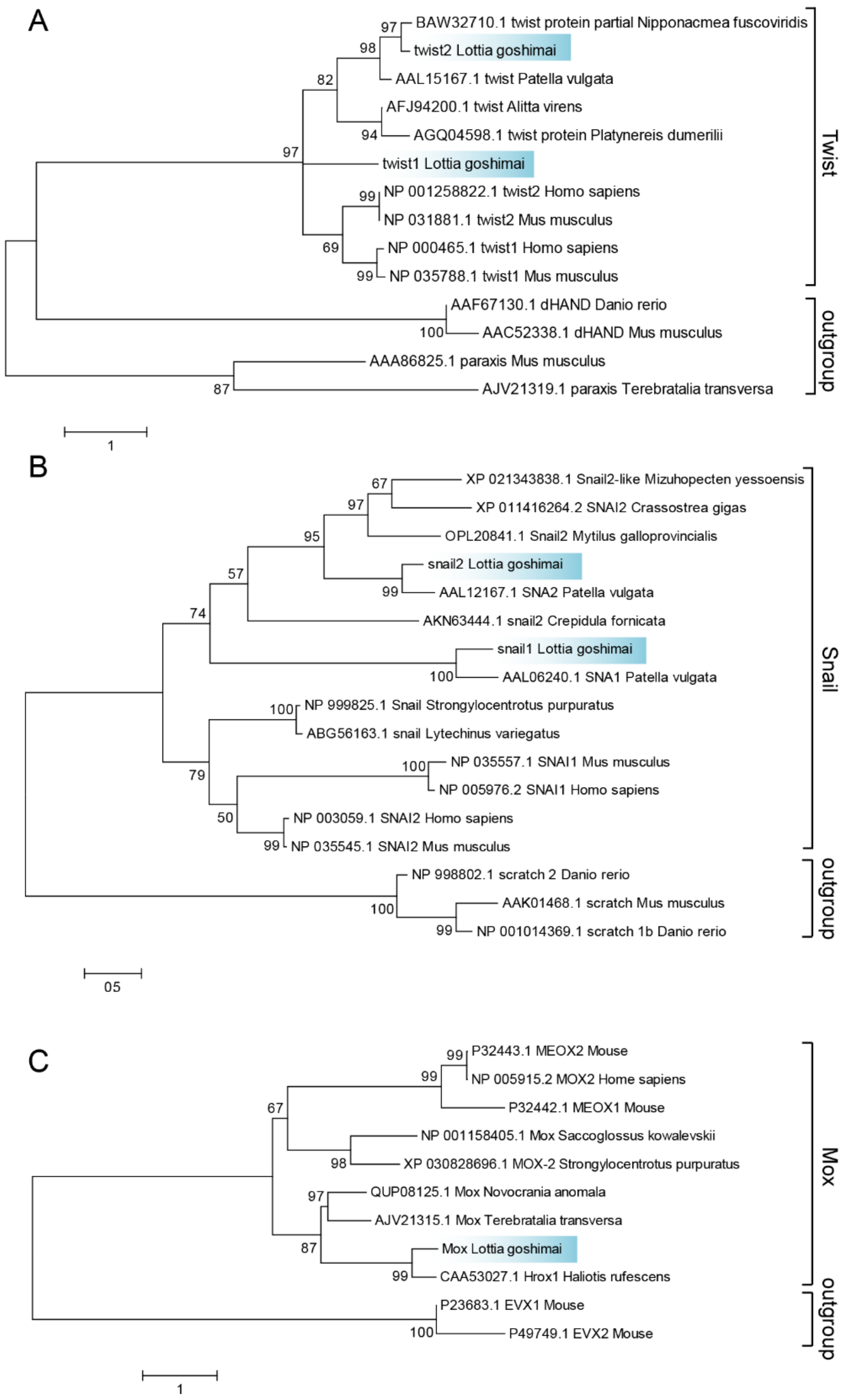

Fig. S1 ML tree of mesoderm marker genes: *twist* (A), *snail* (B), and *mox* (C). JTT+G model was estimated to be the best fitting model for **A** and **B**, and JTT+G+I+F model was estimated to be the best fitting model for **C.** Each node was calculated from 1000 bootstrap replicates.

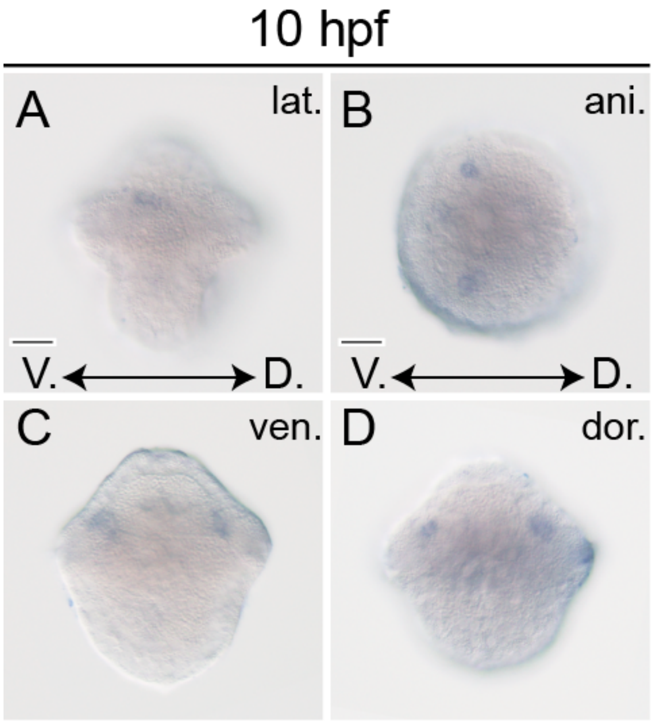

Fig. S2 The expression pattern of *twist2* at 10 hpf. No WMISH signals were detected in earlier samples (4-8 hpf). The bars represent 25 μm.

Table S1. Primers used in WMISH

Gene	GenBank Accession No.	Primers (5'-3')
twist1	OL457652	forward: ACAGTTATCATCATTCGTTAGA<br>reverse: ACCTTCCATTCTCCATACT
twist2	OL457653	forward: CAAGTTCCGATTATCCGATT<br>reverse: TCAGTGTCCATTCATAGCC
snail1	OL457656	forward: TCACTTCCACCAATCCAA<br>reverse: CTCTCCAGTATGTGTTCTAATATG
snail2	OL457654	forward: GCTTCCATCTCCATCTGA<br>reverse: GTATGTGTTCTTATATGTCCTTGT
mox	OL457655	forward: ACGGTTCTTATCCATCCAA<br>reverse: CCATATCATCTTCACAATCCA
